# Mapping co-ancestry connections between the genome of a Medieval individual and modern Europeans

**DOI:** 10.1038/s41598-020-64007-2

**Published:** 2020-04-22

**Authors:** Manuel Ferrando-Bernal, Carlos Morcillo-Suarez, Toni de-Dios, Pere Gelabert, Sergi Civit, Antonia Díaz-Carvajal, Imma Ollich-Castanyer, Morten E. Allentoft, Sergi Valverde, Carles Lalueza-Fox

**Affiliations:** 1 Institute of Evolutionary Biology (CSIC-Universitat Pompeu Fabra), 08003 Barcelona, Spain; 2 Department of Evolutionary Anthropology, University of Vienna, Vienna, Austria; 3 Departament d’Estadística, Facultat de Biologia, Universitat de Barcelona, 08028 Barcelona, Spain; 4 Grup de Recerca d’Arqueologia Medieval i Postmedieval (GRAMP-UB), Departament d’Història i Arqueologia, Facultat de Geografia i Història, Universitat de Barcelona, 08001 Barcelona, Spain; 5 Lundbeck Foundation GeoGenetics Centre, The Globe Institute, University of Copenhagen, 1350 Copenhagen, Denmark

**Keywords:** Evolution, Molecular biology

## Abstract

Historical genetic links among similar populations can be difficult to establish. Identity by descent (IBD) analyses find genomic blocks that represent direct genealogical relationships among individuals. However, this method has rarely been applied to ancient genomes because IBD stretches are progressively fragmented by recombination and thus not recognizable after few tens of generations. To explore such genealogical relationships, we estimated long IBD blocks among modern Europeans, generating networks to uncover the genetic structures. We found that Basques, Sardinians, Icelanders and Orcadians form, each of them, highly intraconnected sub-clusters in a European network, indicating dense genealogical links within small, isolated populations. We also exposed individual genealogical links -such as the connection between one Basque and one Icelandic individual- that cannot be uncovered with other, widely used population genetics methods such as PCA or ADMIXTURE. Moreover, using ancient DNA technology we sequenced a Late Medieval individual (Barcelona, Spain) to high genomic coverage and identified IBD blocks shared between her and modern Europeans. The Medieval IBD blocks are statistically overrepresented only in modern Spaniards, which is the geographically closest population. This approach can be used to produce a fine-scale reflection of shared ancestry across different populations of the world, offering a direct genetic link from the past to the present.

## Introduction

Many studies have demonstrated that human population genetic structuring in Europe correlates with geography; for instance, a two dimensional representation of the genetic variation with principal component analysis (PCA) essentially mirrors a geographical map of Europe^1,2^. Several ancient DNA (aDNA) studies have shown that the overall genetic structure was shaped by three ancestral and over-imposed genomic components respectively deriving from the Mesolithic hunter-gatherers, the Early Neolithic farmers, and the steppe nomads that entered Europe from the East around 5,000 years ago^3–7^. However, it is expected that the genetic homogenisation of the European populations during the last two millennia complicates our ability to discern subtle changes in ancestry by using some common population genetic tools.

Complementary to these analyses, the distribution of so-called identity by descent (IBD) genomic stretches, which are co-inherited genetic segments delimited by recombination events, can provide information on more recently shared ancestry among individuals^8–10^. Such genomic block characterization in current populations has demonstrated the presence of co-ancestry across geographically distant Europeans shared over the last few thousand years, and revealed more recently shared co-ancestry in neighboring populations^11^. Nevertheless, most IBD blocks are not expected to be recognizable after a few hundreds of years because they are being broken by recombination during meiosis. Since the far majority of ancient human genomes sequenced to date are >2,000 years old and only few of them are sequenced at high coverage, the IBD analytical framework seems incompatible with the time scale and data quality offered by most published ancient DNA studies.

To explore genealogical IBD structure among populations, we used a reference dataset of genome-wide data from modern European individuals and we sequenced the genome of a 600 year-old Medieval skeleton from Barcelona (Spain) to high coverage. We subsequently used methods of graph theory to visualize the ancestry connections both among modern Europeans, and between modern Europeans and this Medieval individual. Networks can be used to analyze and visualize interactions between elements^12^ for example between phages and their bacterial hosts^13^ or other ecological and evolutionary interactions. Here, we use a network framework to infer genomic similarity, measured as shared ancestry between individuals, an approach that has previously been used on modern human genomic data^14–16^. By combining aDNA data with the analysis of genomic blocks we establish, for the first time, the direct genealogical links between present-day people and a historical ancestor.

## Results

### IBD analysis of modern populations

**Figure 1:**
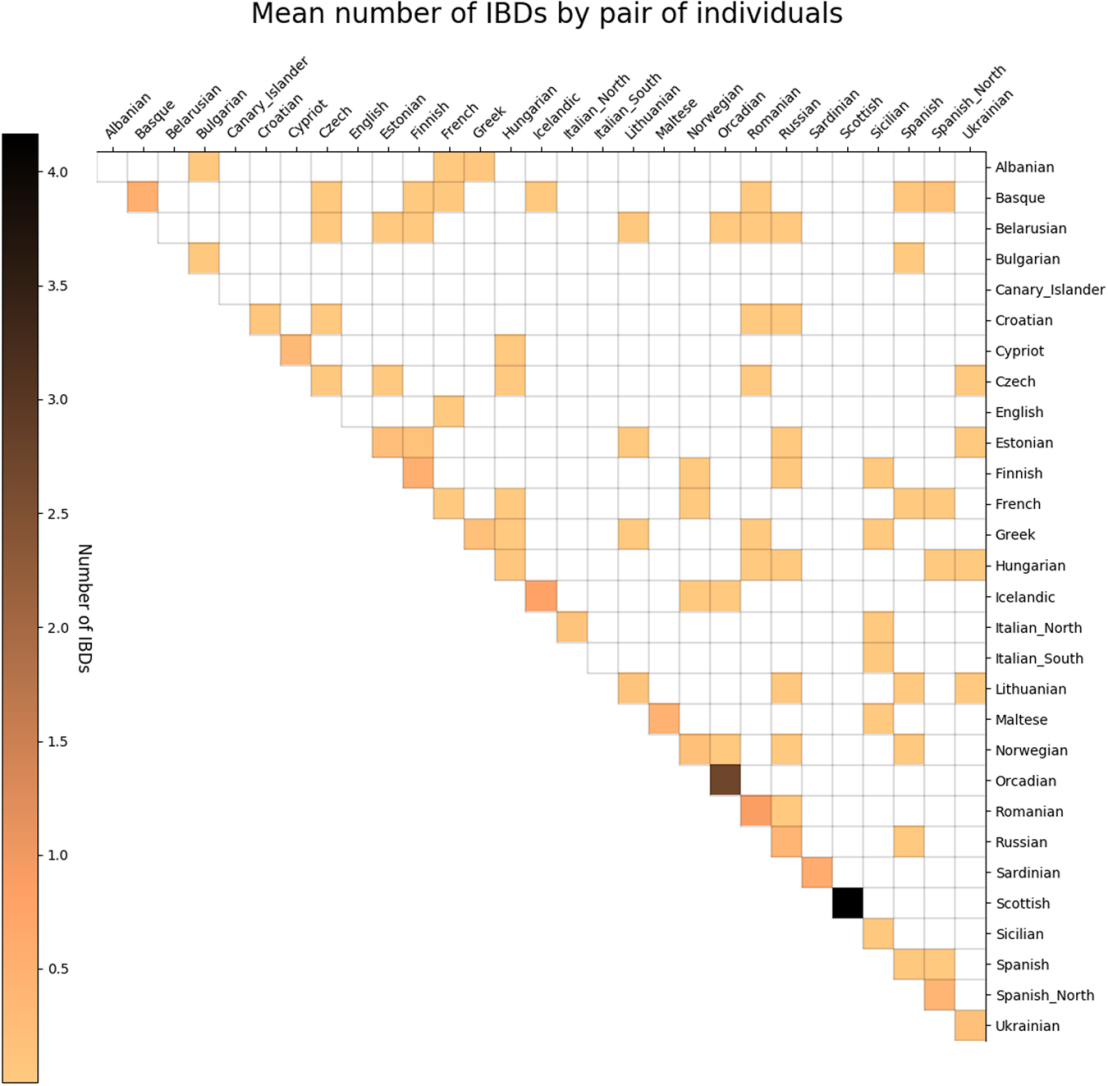
Mean number of IBD segments (>6 cM) shared by pairs of individuals belonging to two given populations in the modern European dataset. It can be observed that most of IBD blocks tend to fall within and not between populations, with some exceptions.

To explore the general genetic structure of European co-ancestry, we filtered the Human Origins dataset^17^ to analyze 429 individuals (Table S1) and 495,239 SNPs. We detected 1,249 genomic IBD blocks longer than 6 cM among pairs of European individuals summarized as 1,014 intra population and 235 inter population pairs (Table S2). Because these 429 individuals can be combined to generate 5,403 intra and 86,403 inter population pairs, it implies that IBD blocks are identified 70.2 times more frequently within than between populations (1,014/5,403 = 0.1895 IBD blocks per pair of individuals vs 235/86,403 = 0.0027 IBD blocks per pair of individuals, respectively) (Fig. 1); interestingly, a similar observation was made with a different European human genomic dataset (POPRES)^11^. Some of the inter population connections we observed are plausible in the light of recent history such as IBD tracks shared among individuals from Estonia, Russian, Lithuania and Finland or IBD tracks shared among inhabitants from Iceland, Orkney Islands and Norway.

**Figure 2:**
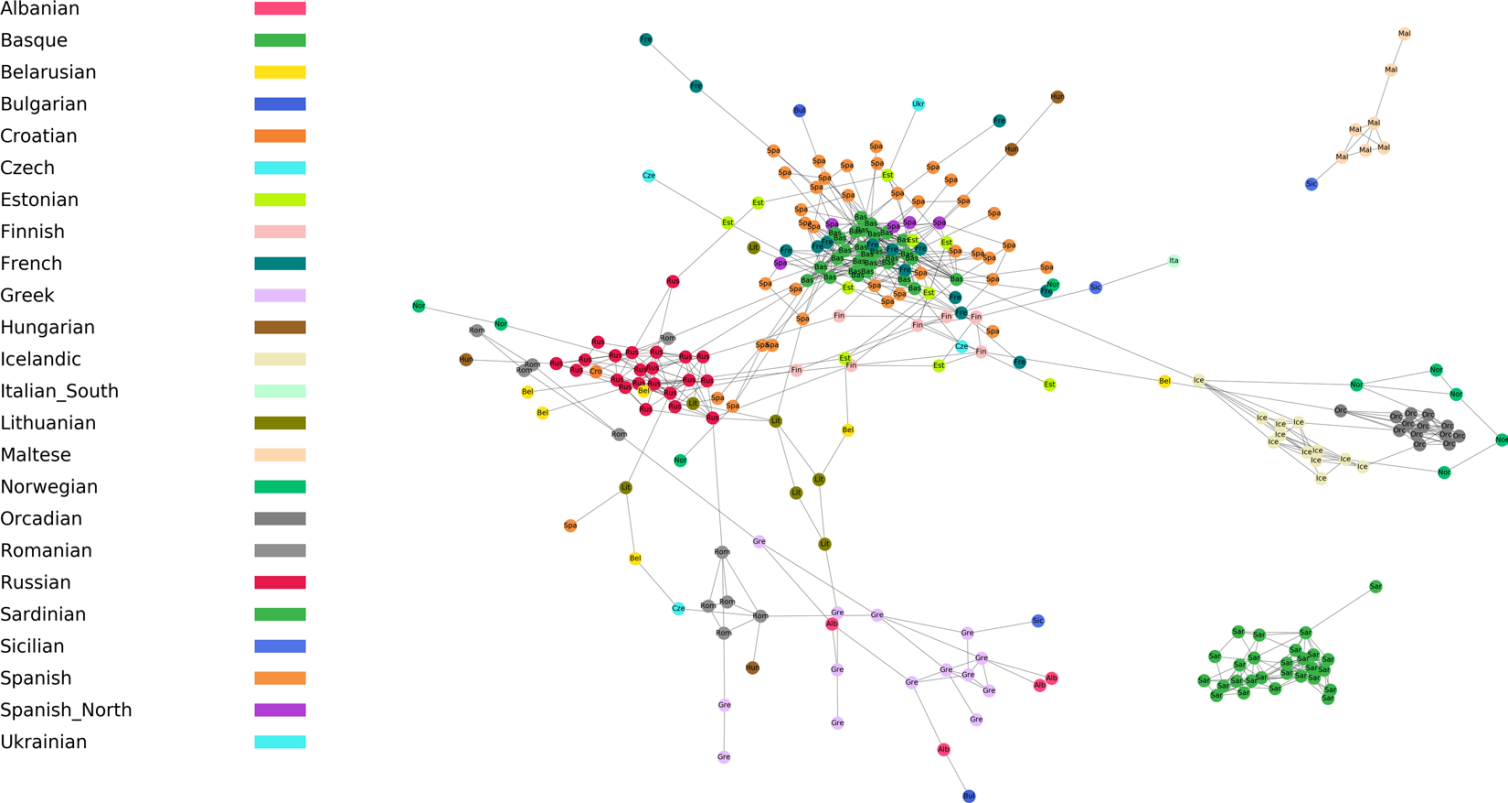
Network of identity by descent (IBD) blocks longer than 6 cM shared between present-day Europeans (nodes). There is a link between any pair of individuals sharing at least one IBD segment. Individuals are labeled according to the population of the Human Origins. Isolated IBD pairs of individuals are not represented for clarity, with the exception of Maltese and Sardinians.

We subsequently used a network representation of the IBD block distribution (Fig. 2) similar to the approach previously applied for modern Europeans and African American exomes^18^. Individuals in the plot differ markedly in their connectivity, with some connected by IBD blocks to many individuals while others, in the peripheral branches of the network, being connected only to a single individual. This network displays community structure, i.e., the occurrence of groups of nodes (or modules) that are more densely connected internally than they are with the rest of the network. Individuals belonging to small and historically isolated populations such as Basque-speakers, Orcadians, Sardinians and Icelanders tend to constitute such densely internally connected modules. Other potential modules such as that observed among individuals from Russia are likely explained by biased sampling for example owing to endogamy in a small and isolated community.

A total of 109 individuals are disconnected from the main network. Remarkably, all Sardinian individuals appear genetically isolated from the rest of the continent, an observation that is in agreement with ancient genomic studies where Sardinians are shown to largely preserve the genetic legacy of early Neolithic farmers^4–6,19^. The Maltese individuals display a similar situation, with seven of them forming their own cluster together with a single Sicilian individual.

**Figure 3:**
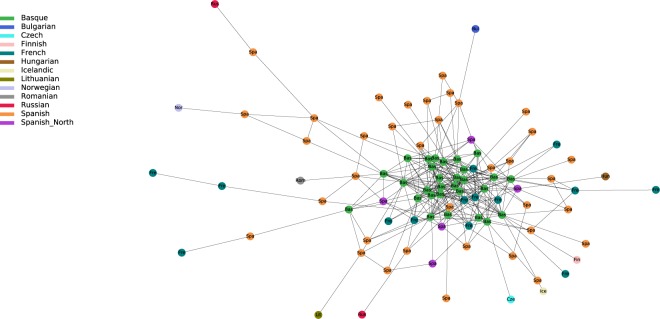
Regional network of present-day Basques. It includes Basques and individuals sharing IBD blocks with Basques.

**Figure 4:**
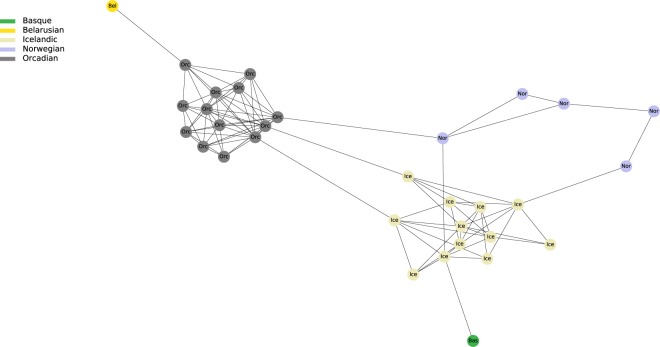
Regional network of present-day North Atlantic Europeans. It includes Icelandic, Norwegian and Orcadian and individuals sharing IBD blocks with those populations.

**Figure 5:**
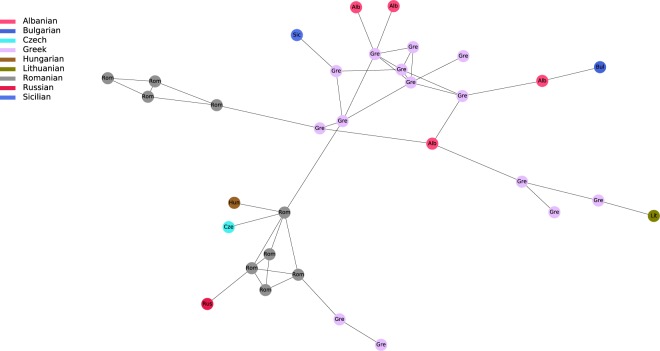
Regional network of present-day south Eastern Europeans. It includes Greeks, Romanians, Albanians and Bulgarians and individuals sharing IBD blocks with those populations.

By plotting IBD connections within and among specifically selected geographic regions, we can see diverging co-ancestry patterns, indicative of differences in the population demographies during the last few centuries. For instance, the Basque region shows a tight clustering of Basque individuals surrounded by a more disperse clustering of Spanish and French individuals (Fig. 3). The so-called “Spanish-North” group, which derives from Alava -a region where Basque language was still spoken in historical times- is located in an intermediate position between Basque speakers and Spanish individuals. Individuals from Iceland, Norway and Orkney islands present almost exclusively intra-population connections but with some links between them. This is in agreement with a presumed settlement of Iceland from the Atlantic North^20^ (Fig. 4). By contrast, Southeastern Europe shows a higher degree of mixed connectivity with a number of interpopulation connections to different countries. This suggests a higher level of population heterogeneity, possibly resulting from more recent population movements (Fig. 5).

### IBD analysis with a Medieval individual

To test for the presence of co-ancestry links back into the historical past, we selected a Medieval skeleton for genome sequencing. The individual numbered T-145-2 (Fig. S1), was from the mid-XIVth century^21^ and was excavated at the Medieval village of L’Esquerda near Roda de Ter (North of Barcelona, Catalonia). This village was abandoned during the Black Death Plague epidemic^22,23^. We extracted aDNA from the otic capsule of the petrous part of the temporal bone^24^, constructed a double stranded library, and generated a total of 1,103,685,282 DNA reads. After mapping the reads to the hg19 human reference genome, the putative endogenous human DNA content was calculated as 62.3% of the sequences. After removing duplicated reads and passing quality filtering steps, 543,173,362 unique reads remained, yielding 11.3x depth of coverage for this ancient genome. The C-T/G-A postmortem damage at the 5′ and 3′ ends of the reads, which is a signal of DNA authenticity, was 26.4% and 17.3%, respectively (c). Contamination was estimated to be 1.8% by looking for discordant nucleotides at defining positions of the mtDNA K1C1 haplotype observed for the T-145-2 individual. With a method combining deamination patterns and the fragment length distribution^25^, we obtained a similarly low contamination estimate (0.5%–2.5%). All these results testify to authentic ancient DNA molecules with a negligible proportion of modern DNA contamination,

To our knowledge, only fifteen other ancient European genomes have been sequenced to a higher coverage: a Scandinavian Mesolithic individual (57.8x)^26^, a Mesolithic individual from Loschbour (22x) and a Neolithic individual from Stuttgart (19x)^4^, Hungarian Bronze Age and Neolithic individuals (21x and 22x, respectively)^24^, a Copper Age individual from Spain (13x)^27^, an Iron Age individual from Hinxton (11x)^28^, six Longobards (12.86–14.48x)^29^ and two early Icelandic individuals (12.9x and 30.7x)^20^. With the current genomic coverage of the Medieval L’Esquerda individual, it was possible to generate accurate genotypes calls^30^ that were subsequently merged with SNPs from the Human Origins panel.

**Figure 6:**
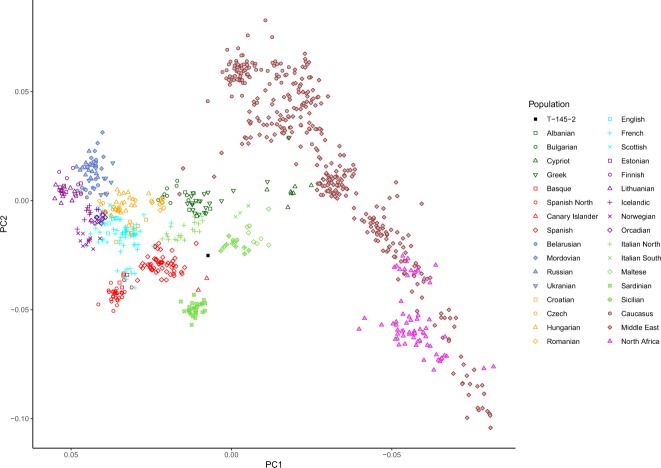
Principal Component Analysis (PCA) with Human Origins’ modern European, Middle East and North African individuals, including also the Medieval individual. Labels for Middle East and North African populations have been grouped for clarity. North Africans have been projected to avoid distorting the European distribution.

In a principal component analysis (PCA) constructed with modern Europeans, Near Easterns and North African individuals as reference, our Late Medieval individual is placed in an intermediate position in the representation of the first two axes, genetically close to modern day individuals from North Italy, Spain and France (Fig. 6). In the ADMIXTURE analysis (K = 4) four genomic components - represented by the ancestry of European hunter-gatherers, Early Neolithic farmers, Late Neolithic steppe nomads, and North African individuals - can be detected (Figs. S3 and S4). We observe that the Medieval individual has more North African ancestry than the average observed in modern Iberians. This would also explain its position in the European PCA; the same trend is seen in other early Medieval individuals from the same region^7^, suggesting there was a subsequent dilution of this North African component in more recent times.

**Figure 7:**
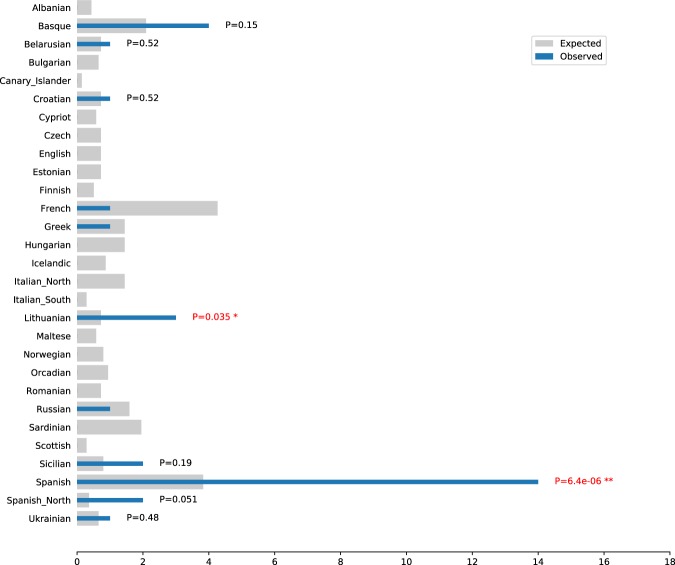
Observed and expected IBD segments between the medieval individual and individuals of each population. Expected values are generated assuming no correlation between the population to which the individuals belong and its probability of presenting an IBD segment with the Medieval individual. The distribution of IBD segments for each population was assumed to follow a binomial distribution. The p-values were adjusted with a Benjamini-Hochberg procedure to control for false discovery rate.

We then identified IBD blocks based on 369.859 SNPs that were shared between modern Europeans and the Medieval genome. We found a total of only 31 IBD tracks longer than 2 cM (Table S3); 19 of them (61,3%) were shared with individuals from the Iberian Peninsula. As expected, a decreasing number of IBD blocks were found by increasing the length threshold: seven IBD blocks >3 cM and only one >5 cM (Table S3). This single relatively long IBD was shared with a Catalan individual, thus coming from the same geographical region as T-145-2. No IBD blocks >6 cM were found. Although IBD blocks shared with our Medieval individual show a restricted geographical distribution (i.e. individuals labeled as Spanish, Spanish North and Basques in the Human Origins panel), there are still indications of surprisingly long-distance connections to some modern individuals. For instance, there are three IBD segments shared with three Lithuanian individuals, although we cannot exclude that some of these could be false positives. Moreover, after an statistical correction, the only significant overrepresentation of IBD blocks between the Medieval individual and modern populations is observed with inhabitants from Spain (Fig. 7).

## Discussion

Generally, European IBD blocks longer than 4 cM derive from common ancestors living 500–1500 years ago or even more recently^11^, which is a period roughly contemporaneous to our Medieval individual. The network based on >6 cM genomic blocks in modern Europeans uncovers some interesting genealogical features that are not evident in the commonly applied population genetic methods such as PCAs or ADMIXTURE analyses. For example, these networks allow us to discriminate genetically between the demographic histories of small and possibly endogamous populations such as Icelanders or Orcadians versus large and highly dispersed continental populations that constitute the backbone of the network. A certain over-sampling of isolated groups or particularly “interesting” sub-populations (such as Basques or Sardinians) can partially explain the modules, but reducing the number of individuals from these groups does not change the overall pattern because these isolated populations are generally connected to the rest of the network by none or very few IBD links.

On the contrary, a more diffuse scattering of some populations across the network is informative of high genetic heterogeneity and more blurred co-ancestry links. For instance, individuals from Greece and the Czech Republic are virtually dispersed along different branches, suggesting that they derive from regions where large population movements and admixture took place during the least few centuries.

The network also unravels individual connections that are unlikely to be exposed with other analytical approaches. For instance, in the European network (Fig. 2) we found a cluster of seven Maltese individuals also connected to one Sicilian. This could reflect the XIth century CE invasion of the island by Normands from Sicily. This direct link between individuals is not observed in population genetic analyses such as PCA and Admixture where Maltese and Sicilians cluster with their own respective populations, with whom they share most of their overall ancestry (Fig. 6). We also observed an Icelander sharing one IBD segment with a Basque individual (Fig. 4). Basque whaling ships were common in the Icelandic Westfjords during the XVIIth century CE^31^ and they even developed a Basque-Icelandic pidgin language for trading purposes^32^. It is not implausible that this signal derives from a child conceived by an Icelandic woman and a Basque sailor dating back to that period. Again, modern Icelanders, including this individual, cluster together and far away from Basques in traditional analyses based on overall ancestry (Fig. 6). In-depth genealogical and genetic analyses of modern Icelanders are required to confirm this finding but our results underline the potential for IBD approaches to unravel direct genealogical connections across large geographical areas. The method can also visualize larger demographic trends, for example represented by the network of south Eastern Europe that suggests a high level of population intermixing and a more complex demographic history in the last hundreds of years.

The Medieval individual shows a reduced number of IBD blocks and with slightly shorter lengths than those shared between random modern European individuals from different populations (Figs. S5, S6). This is expected because of recombination breaking down IBDs over time. The pattern of genomic block-sharing between the Medieval individual and modern European populations points to a restricted geographical clustering, with an increased number of co-ancestry links to present-day Iberians. This is essentially an observation of isolation by distance induced by shared ancestry and some degree of isolation among Iberians in the last hundreds of years. A similar observation about the apparent isolation of Iberian populations since the Middle Ages was made with a different human genomic dataset and similar number of SNPs (POPRES)^11^. Moreover, the Late Medieval individual displays a limited but significant number of geographically distant relationships that point to more ubiquitous co-ancestry. This confirms previous inferences based on genomic data from modern populations that showed presence of more diffuse co-ancestry links extending further back in time^11^. We note that some long IBD blocks could potentially be the product of concatenated, shorter IBD blocks and thus constitute false positives suggesting distant connections^11^. To reject such potential false positives is not trivial and would require additional sequence data from these specific individuals that could provide additional, intermediate SNPs across our detected IBD blocks.

Nevertheless, both types of ancestry -ubiquitous and geographically-proximate - are expected to be present in the IBD results when combining ancient and modern genetic data. The IBD blocks shared between a Medieval and modern Europeans are shorter but seem to be also more geographically clustered compared to those found among present-day individuals, with the exception of the previously mentioned isolated populations. If additional Medieval, high coverage genomes become available in the future, it is likely that more locally restricted IBD blocks could be identified across Europe.

The power of our approach to uncover individual genetic affinities in otherwise homogeneous populations is further emphasized when comparing with a PCA analysis that includes our Medieval genome. With this standard analysis, it was not possible to attribute a specific population affinity of our individual that was occupying an equidistant position between modern individuals from Spain, Sicily and North Italy. However, the IBD analysis clearly placed it among modern Spanish individuals. Remarkably, no IBD tracks are shared between the Medieval and individuals from North Italy, and only two with Sicilians, despite the apparent proximity of these populations in the PCA. This constitutes an example of the power of this approach to detect micro regional affinities among populations that otherwise are quite similar genetically.

With the current level of productivity in ancient genomic research, many more individuals from the recent historical past will be sequenced to high genomic coverage in the near future. This will allow us to extend this methodological framework to an increasingly large genomic population dataset of ancient and modern people^33^. This approach will serve not only to uncover individual ancestry links, but it could also unravel the origins and the spread of mutations subjected to positive selection, because this process should preserve longer genomic blocks than expected under a process of random recombination^34^. In essence, such data will help visualize an extended family tree with a vast, interconnected and complex network, linking the past population genetic landscape with the present one.

## Material and methods

### The site

L’Esquerda is an archaeological site placed in an area of 12 hectares on cliffs overlooking a narrow meander of Ter river, near Roda de Ter (North of Barcelona). Due to its privileged geographical position it has been continuously occupied from the Late Bronze Age to the Middle Ages^22^. The Visigothic settlement was temporarily abandoned during the Muslim occupation until the Carolingian times, when it was settled again, in parallel to the Frank conquest of Girona in 785 C.E. The Medieval village grew up around the church of Sant Pere de Roda, built in the XIth century over a previous, smaller church. A walled area around the church was destined for cemetery, where three main stratigraphic layers can be observed: a basal one from the Visigothic and Carolingian periods, an intermediate one with slab-stone burials dated between the XIth and the last XIIIth century and a superficial one from the final occupational period of the settlement. Despite L’Esquerda was destroyed and abandoned in 1314 for a new location close to the river, the final use of the burial area consists on communal graves dated to the first two-thirds of the XIVth century associated to the epidemics of 1348 and subsequent years^23^.

The individual analyzed, labeled T-145-2, corresponds to a young (15–16 years-old) female that was excavated in the seasons 2009–2010 in one of the XIVth century’s graves, along with another adolescent and an adult male that were buried simultaneously. A right petrous bone was selected for DNA extraction, due to better chances of DNA preservation^24^.

### DNA extraction and sequencing

The petrous portion of the temporal bone was sliced open using an electric diamond-coated cutting blade allowing us to remove and crush the otic capsule for DNA extraction^35^. The DNA was extracted using a silica-in-solution method optimized for retaining short and degraded DNA molecules^6^. First, a 15-min enzymatic pre-digestion step was implemented to reduce the amount of exogenous DNA^36^. The samples were then incubated for 24 hours at 45 °C in 5 ml digestion buffer containing 4.7 ml 0.5 M EDTA, 50 μL Proteinase K (0.14–0.22 mg/ml, Roche), 250 μL 10% N-Laurylsarcosyl, and 50 μL TE buffer (100x). The solution was spun down and the supernatant transferred to a 50 ml tube, where it was mixed with 100 μl silica suspension and 40 ml binding buffer, prepared as in Allentoft *et al*.^6^. After 1 hour of incubation, the supernatant was removed and the pelleted silica was re-suspended in 1 ml binding buffer, spun down and washed twice with 1 ml 80% cold ethanol. Finally, the DNA was eluted in 80 μl EB buffer (Qiagen). Extraction blanks were also included. Next, 2*20 μl of DNA extract was prepared as blunt-ended, double-stranded libraries using Illumina-specific adapters and the NEBNext DNA Sample Pre Master Mix Set 2 (E6070) kit, as described previously^6^, except that we here used the KAPA HiFi HotStart Uracil + ReadyMix (KAPA Biosystems, Woburn, MA, USA) in the amplification step. The two index-amplified DNA libraries were purified and quantified on an Agilent Bioanalyzer 2100. The DNA extraction and library preparation (pre-amplification steps) were conducted using strict aDNA guidelines in a sterile clean lab at Centre for GeoGenetics at the Natural History Museum of Denmark. The libraries were sequenced (80 bp, single end sequencing) on an Illumina HiSeq. 2500 platform at the Danish National High-throughput DNA Sequencing Centre.

### Mapping Procedure

Sequencing-adapters were trimmed with Cutadapt 1.3^37^. The clipped reads were mapped against the human reference genome (hg19) and the revised Cambridge Reference Sequence (rCRS)^38^ using BWA aln^39^ setting no seeding, no read trimming, an edit distance of 0.01 and a gap open penalty of 2. Afterwards, duplicated reads were removed with Picard tools 2.18.6^40^. Finally, unique reads were filtered with SAMtools 1.6^41^ keeping only those with mapping qualities over 30. The mapped and filtered reads were analyzed with MapDamage 2.0.8 to determine the post-mortem aDNA damage pattern^42^. Because the final sequences present a deamination percentage of 26.4% and 17.3% in the 5′ and 3′ ends respectively (Fig. S2), which could affect the variant calling, we trimmed 5 bases at each end using trimbam 1.0.13^43^.

### Contamination estimates

The average level of DNA contamination was estimated by genotyping the mitochondrial DNA haplogroups with Haplogrep2^44^ and calculating the ratio of discordant reads with a homemade script. Modern mitochondrial contamination was estimated using *schmutzi*^25^.

### Variant Calling

Genotypes were called with the Genome Analysis Tool Kit (GATK) v3.7, as previously described^45^, using UnifiedGenotyper and a correction for the observed contamination (–contamination_fraction_to_filter 0.02232),–output_mode EMIT_ALL_CONFIDENT_SITES (this option is important to ascertain which nucleotide position displays the reference allele), the Human Genome 37/19 as the reference. We genotyped 616,938 positions present in the Human Origins dataset^17^. Subsequently, variants with base qualities below 30 and genotype quality below 20 were discarded from the called bases by using VCFtools v0.1.14^46^. We decided to filter out those variants displaying a lower coverage than the average depth of coverage of each chromosome. Despite the medium-high coverage of most of the genome, this procedure should in principle be enough for a confident variant calling. Filtered variants were merged with the Human Origins dataset using PLINK v1.9b^47^. Only SNPs present in autosomal chromosomes were used in the analyses.

### Mitochondrial DNA haplogroup and molecular sex assignment

Mitochondrial DNA genome variants were called with Genome Analyses Toolkit (GATK) UnifiedGenotyper^45^, setting the same parameters used in the autosomal variant calling procedure. The mitochondrial haplogroup was assessed with Haplogrep 2^44^. Molecular sex was assigned using the methodology used in^48^.

### Datasets Preparation

For the IBD blocks analysis, we downloaded genotypes for 616,938 SNPs and 433 individuals belonging to 29 European populations, from David Reich Lab datasets web page^17^ (Table S1). We removed a total of 121,699 SNPs that did not fulfill our quality control criteria (genotype missingness <5%, Hardy-Weinberg equilibrium test p-value >1×10-6, MAF > 0). After plotting genotype missingness of the individuals against its heterozygosity (Fig. S7), three individuals with particularly extreme values were removed. Relatedness analysis of pairs of individuals discovered a family relationship and one member of this pair was also removed. The resulting filtered dataset (495,239 SNPs and 429 individuals) was used to study IBD relationships between modern Europeans.

A second dataset was generated adding the ancient individual and containing only those SNPs from the European dataset that were also recovered from the ancient sample. It consisted of 369,859 SNPs and 430 individuals. The Medieval individual presented a high heterozygosity and low number of runs of homozygosity (ROHs) compared to modern individuals (Figs. S8, S9), which could be influenced by some residual *post-mortem* damage or by the merging of heterogeneous data (whole-genome sequence and genotype data). This dataset was used for studying IBD relationships between the Medieval individual and modern Europeans.

### Population genetic analyses

A Principal Component Analysis (PCA) was built, with 495,239 SNPs and present-day 881European, Middle Eastern, Caucasians and North African individuals from the previously described dataset, using Eigensoft^49,50^. The resulting data was plotted using R package Ggplot2^51,52^.

An admixture analysis was performed with ADMIXTURE^53^. We selectedthe same individuals and a dataset of 16 ancient individuals known to be representatives of the main ancestry components of modern Europeans, and our ancient individual T-145-2^17^. We then filtered the dataset by removing SNPs in linkage-disequilibrium (LD) using PLINK 1.9 flag–indep-pairwise with a windows size of 200 SNPs, advanced by 50 SNPs and establishing an r^2^ threshold of 0.4^47^. We performed the ADMIXTURE analysis, using 242,622 SNPs, with K ranging from 2 to 15 and performing 10 replicates for each run. We selected the K = 4 in accordance to the lowest cross-validation mean value^53^ (Fig. S4) that uncovers the four main European ancestry components: Western Hunter-Gatherers, Early European Farmers, Steppe Nomads and Northern Africans^17,53^. We plotted ADMIXTURE results using package pophelper^54^.

### Haplotype Estimation

Phase of genotypes for both datasets was estimated without imputation of unknown genotypes with Beagle 5.0 software^55^. The recombination map and the haplotype reference panel provided in the Beagle publication were used.

### IBD Discovery in modern Europeans

We examined the data for IBD segments between all pair of European individuals using the Refined IBD software^56^ with the parameter “minimum length for reported IBD segments” set at 1 cM. A total of 289,561 IBD segments were identified with 1,523 being longer than 6 cM and, were selected for further analysis.

Within the set of IBD segments longer than 6 cM we studied the distribution of scores, the relationship between length and score and the coverage along the genome to discover potential false IBD blocks. We removed IBD segments that, after visual examination, clustered outside the main distribution with abnormal low scores considering their length. We also removed IBD segments overlapping centromeric and telomeric regions (Fig. S10). After quality control 1,249 IBD blocks longer than 6 cM remained.

### Triangulation Analysis

To assess the validity of the IBD segments longer than 6 cM among modern European individuals, we checked for the transitivity of the IBD relationships between trios of individuals. If individuals A and B share a IBD segment and individual B, in the same chromosome, shares the same IBD segment with individual C, we expect A and C to share it too (Fig. S11). We grouped overlapping IBD segments into clusters. Two IBD segments were considered to overlap when they shared at least one individual and were located in the same chromosome overlapping at least by one pair base. We obtained, as predicted, clusters consisting of trios of individuals sharing IBD segments in a transitive way, together with clusters showing different arrangements (Fig. S11).

We plotted the chromosomes involved in some of the clusters to understand the origin of these clusters and defined some typologies (Fig. S11). We listed all the pairs of overlapping IBD segments longer than 6 cM, classified them into the previously defined typologies and looked for the third member of the expected transitive triangulation Only 3 pairs out of 220 remained unexplained and point to a possible artifact in the IBD discovery process.

### Graphical Representation of the Networks

To visualize the IBD relationships among the different populations of modern Europeans, we coded the estimated IBD segments previously obtained into a network structure where individuals correspond to nodes that are connected if at least one shared IBD region between them exists. We then plotted the resulting global network and some particular groups of populations using the NetworkX Python package, version 2.2^57^.

### IBD Discovery in the Medieval Individual

The dataset containing the European plus the Medieval individual with 369,859 SNPs underwent the same process of IBD blocks discovery described for the European dataset. A total of 242,158 IBD segments were generated with 1,472 of them longer than 6 cM (but none involving the Medieval individual). IBD blocks with score values lower than 4.8 and close to centromeric and telomeric regions were removed. From the 1,164 remaining, 31 IBD blocks between the Medieval and some other European individual longer than 2 cM were selected for further study. We plotted the number of SNPs on each of these IBD blocks vs the score of the software to explore if some IBD stretches could be false positives (Fig. S12); we found a weak (adjusted R^2^ of 0.165, p = 0.0135) but significant correlation between number of SNPs supporting higher scores, but with our selected criteria we could not discard as false positives even the most distant connections.

### Population enrichment

To test if the Medieval individual presents a significant enrichment of IBD segments shared with particular populations, we assumed as a null hypothesis that there was no association between the probability of an individual of presenting a shared IBD block with the Medieval and the population to which that individual belongs to. Consequently, the expected total number of IBD segments shared by the Medieval with a given population should be proportional to the number of individuals of that population according to a binomial distribution (n = number of individuals in the population, p = total number of IBDs/number of individuals in all populations). The observed number of IBD segments with the Medieval in each population was then compared to the predicted binomial distribution. However, the large number of tests performed with the Medieval are likely to increase type I error rates (also the binomial tests are not independent because the finding of IBD between the Medieval and a given population is likely to influence subsequent tests)^58^. In similar cases of block-positive dependence among tests, it has been shown that a best option to control for false discovery rate (FDR)^59^ is to use the two stage Benjamini-Hochberg (TSBH) procedure^60^. We subsequently adjusted the p-values between observed and expected IBD blocks for the TSBH procedure; a nominal type I error rate (5%) was used to estimate the number of true null hypotheses in the two-stage TSBH with R multtest package^61^.

## Supplementary information

Supplementary information.

Supplementary Table S2

Supplementary Table S3
