# Supplementary material for: Mapping co-ancestry connections between the genome of a Medieval individual and modern Europeans
Source: Sci Rep. 2020 Apr 22;10:6843. doi: 10.1038/s41598-020-64007-2 (PMC7176696; doi:10.1038/s41598-020-64007-2)
Supplement: Supplementary file 1 — Supplementary information. [file 41598_2020_64007_MOESM1_ESM.pdf]

Mapping co-ancestry connections between the genome of a Medieval individual and modern Europeans

Manuel Ferrando-Bernal[1+], Carlos Morcillo-Suarez[1+], Toni de-Dios[1+], Pere Gelabert[1,2], Sergi Civit[3], Antonia Díaz-Carvajal[4], Imma Ollich-Castanyer[4], Morten E. Allentoft[5], Sergi Valverde[1], Carles Lalueza-Fox[1*]

[1]Institute of Evolutionary Biology (CSIC-Universitat Pompeu Fabra), 08003 Barcelona (Spain)

[2]Department of Evolutionary Anthropology, University of Vienna, Vienna, Austria

[3]Departament d'Estadística, Facultat de Biologia, Universitat de Barcelona, 08028 Barcelona (Spain)

[4] Grup de Recerca d'Arqueologia Medieval i Postmedieval (GRAMP-UB), Departament d'Història i Arqueologia, Facultat de Geografia i Història, Universitat de Barcelona, 08001 Barcelona (Spain)

[5] Lundbeck Foundation GeoGenetics Centre, The Globe Institute, University of Copenhagen, 1350 Copenhagen (Denmark)

Supplementary Figures

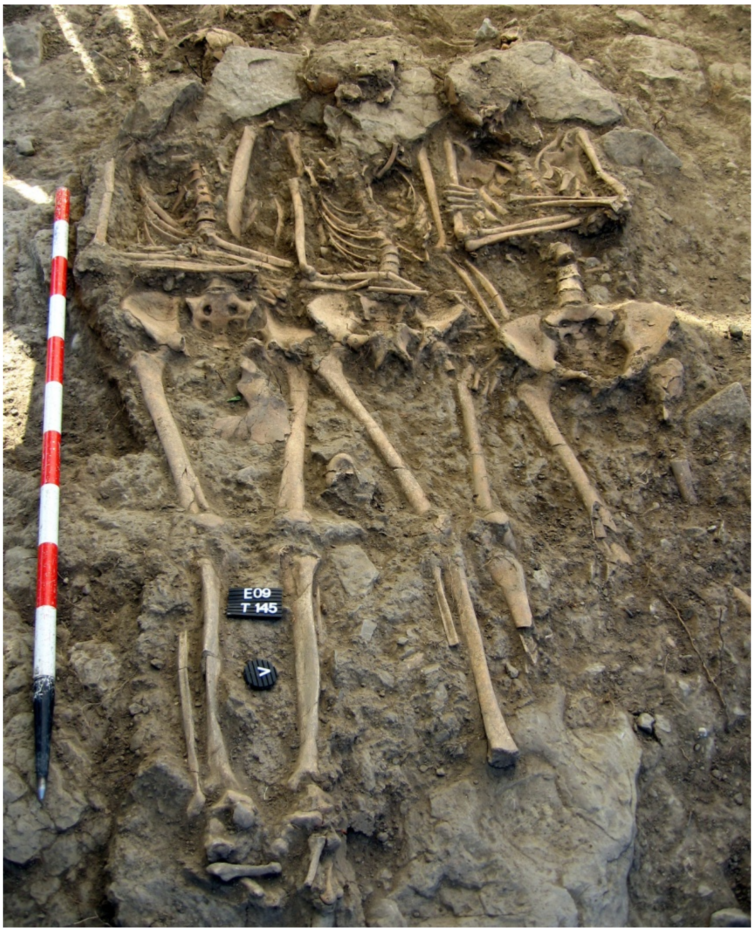

Supplementary Figure S1. The XIVth century skeleton from L'Esquerda, labeled T-145-2, sequenced in this study.

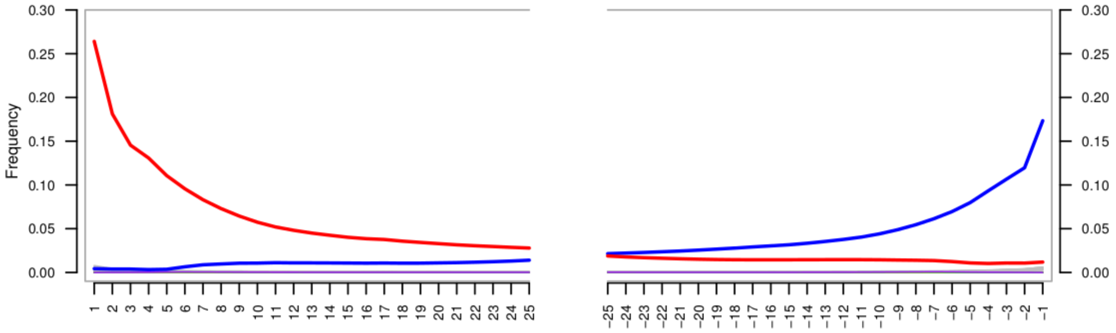

Supplementary Figure S2. *Post-mortem* damage due to ancient DNA deamination processes at the ends of the reads of the T-145-2 sample.

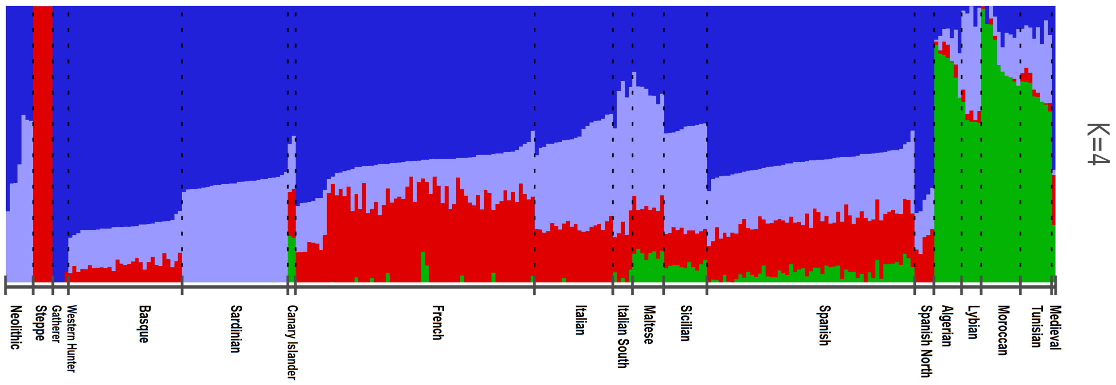

Supplementary Figure S3. ADMIXTURE genetic analysis of the Medieval individual in the context of modern Europeans and North Africans (K=4). Some ancient genomes have been added to show their main contributing ancestry components.

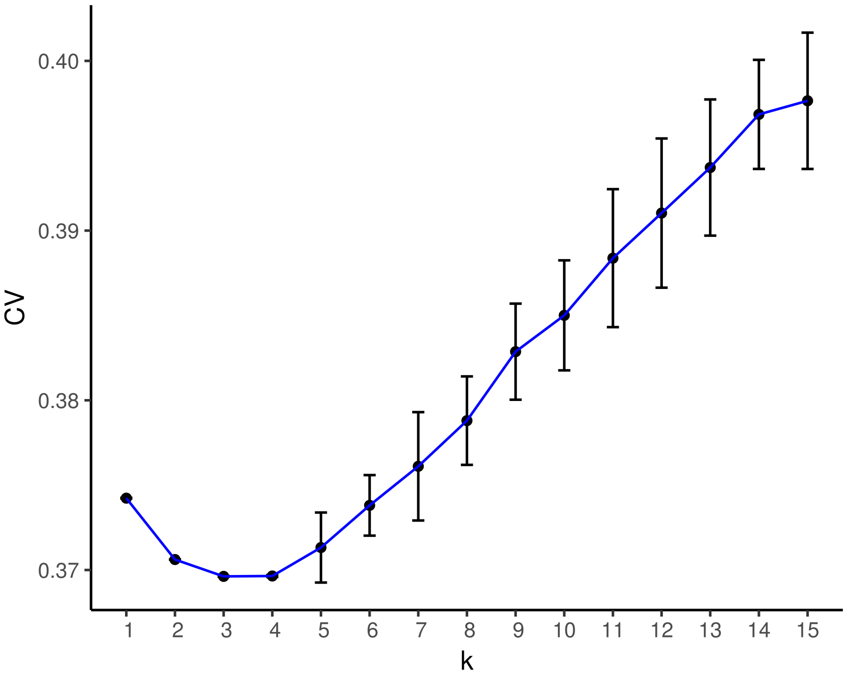

Supplementary Figure S4. ADMIXTURE cross-validation for K values 1-15.

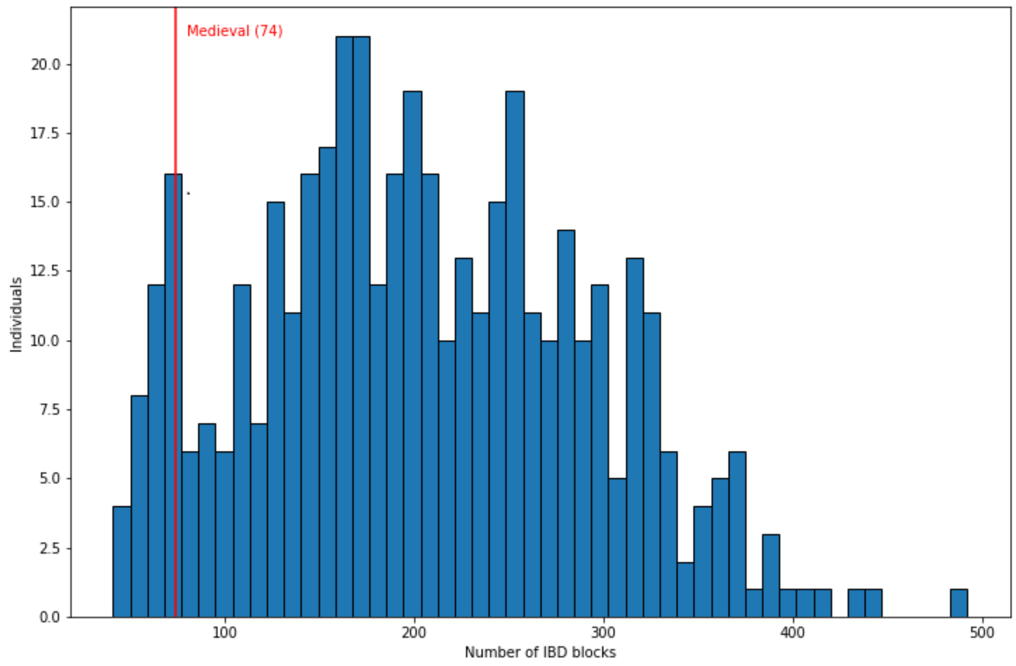

Supplementary Figure S5. Distribution of the number of IBD blocks >1cM that each individual shares with individuals from other populations. In red, the number of IBD blocks shared by the Medieval with individuals of any population.

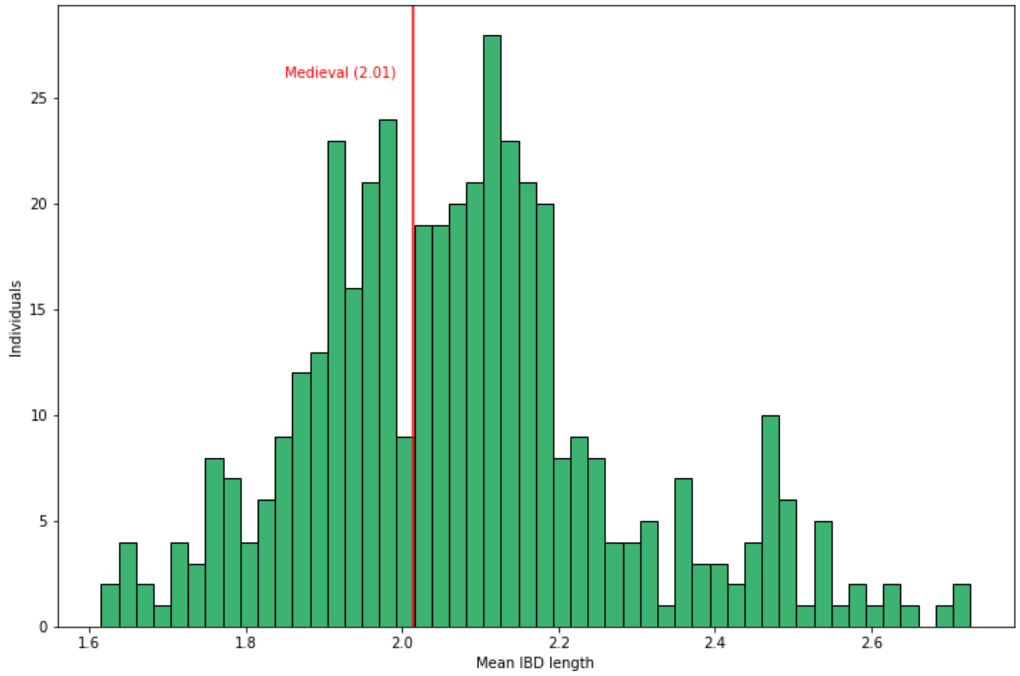

Supplementary Figure S6. Distribution of the mean length of the IBD blocks >1cM that each individual shares with individuals from other populations. In red the mean length of the IBD blocks shared by the Medieval with individuals of any population.

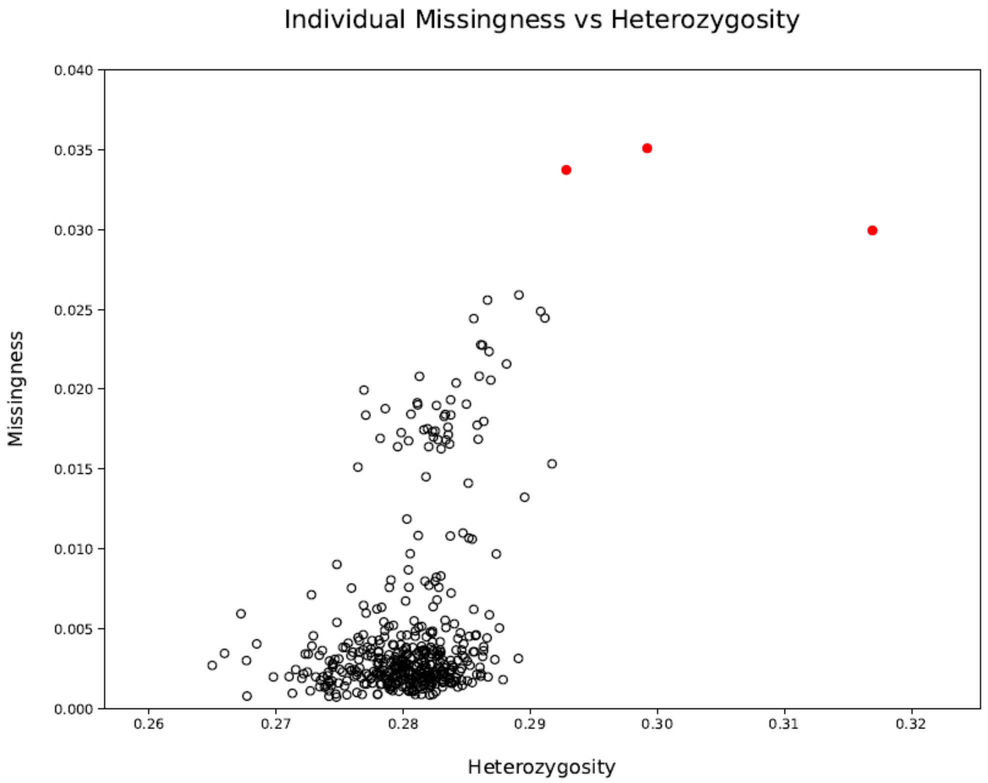

Supplementary Figure S7. Missingness of modern European individuals plotted against their heterozygosity. The three individuals marked in red were removed from the sample.

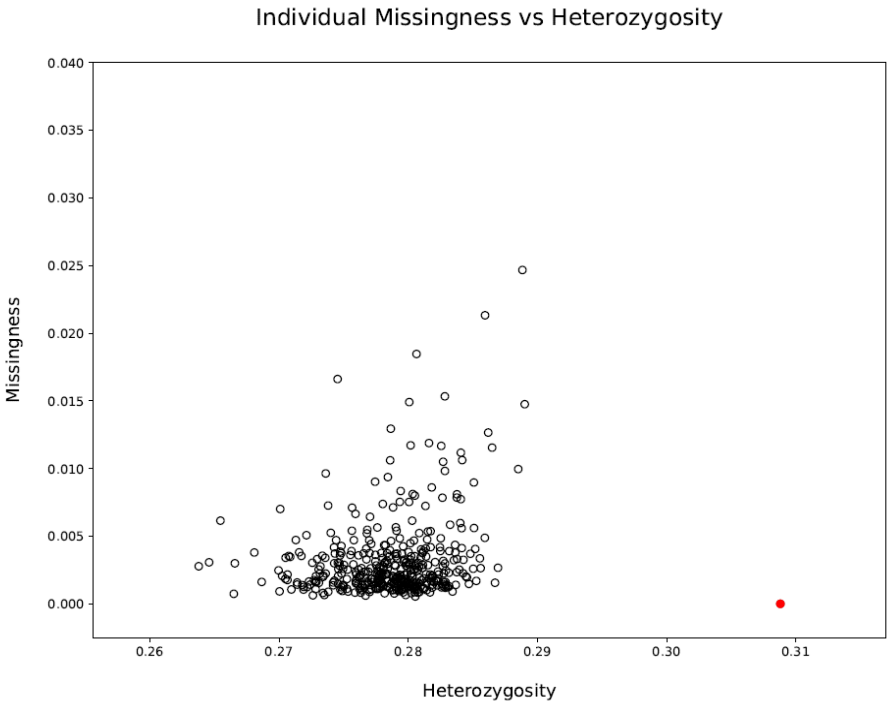

Supplementary Figure S8. Genotype missingness against heterozygosity in the individuals of the IBD dataset. The Medieval individual is marked in red.

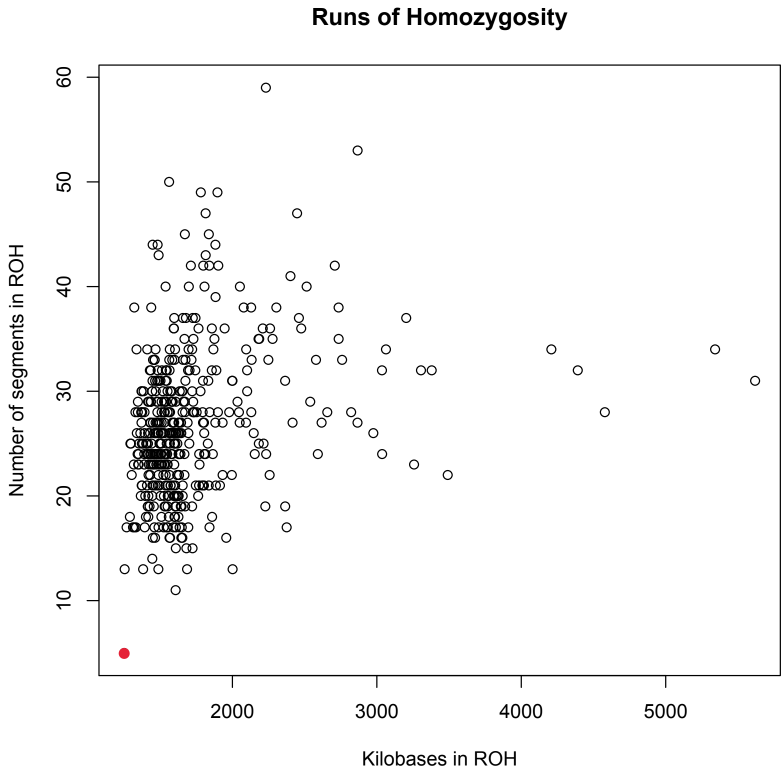

Supplementary Figure S9. Runs of Homozygosity (ROHs) in the dataset. The Medieval individual is marked in red.

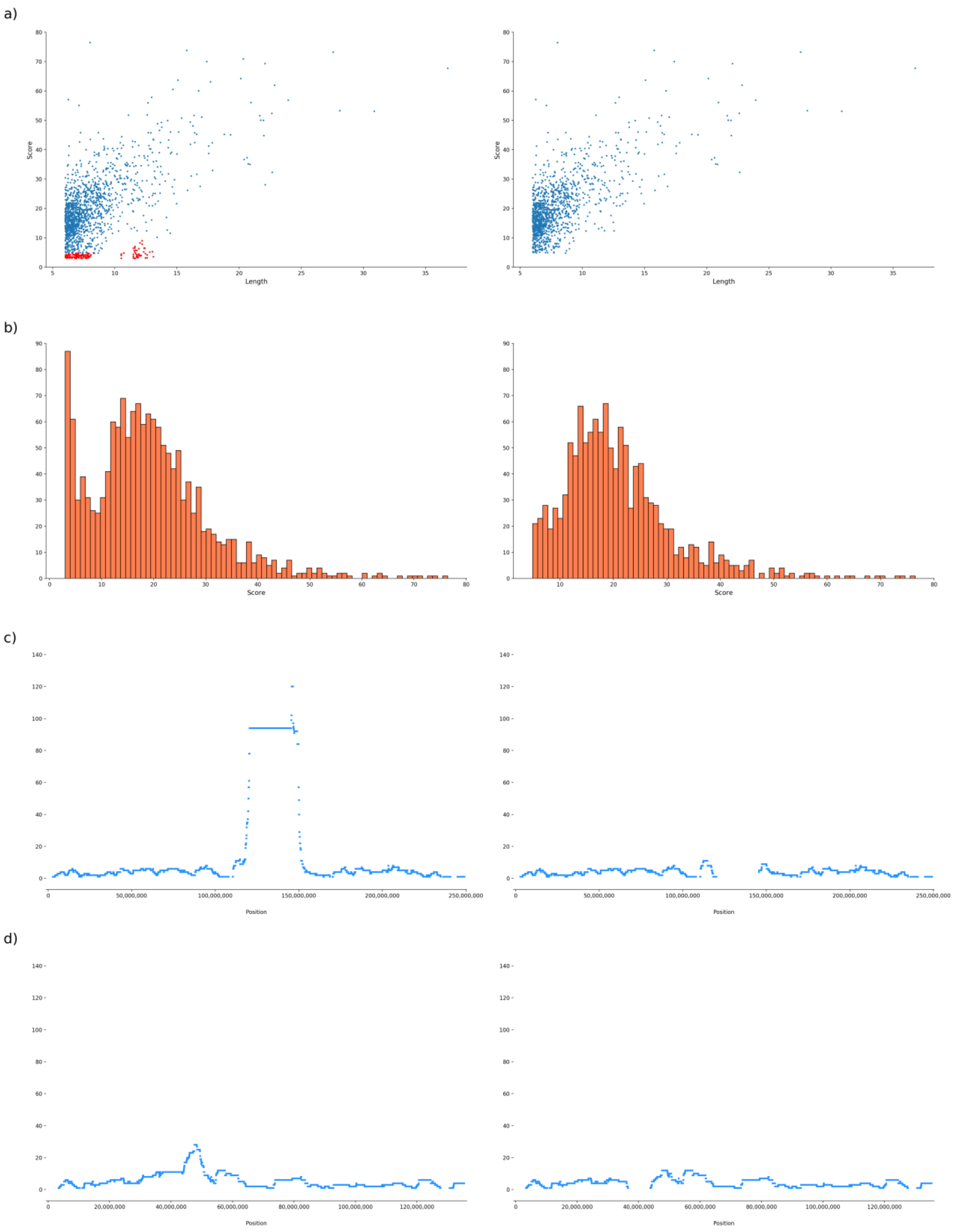

Supplementary Figure S10. IBD segments longer that 6 cM obtained from the European dataset before (left) and after (right) removal of those that did not meet quality control criteria. a) Plot of the score of each IBD segment against its length. Those marked in red were considered to cluster abnormally and were removed. b) Histogram of score values of obtained IBD segments. c) and d) IBD coverage for chromosomes 1 and 10. This corresponds to the number of IBD segments that fall into a particular genomic position.

a)

Chr	Start	End	Ind1	Ind2	Score	Length
1	87,665,502	101,530,528	SouthFrench3326_2	SouthFrench1112_1	32.69	13.18
1	87,932,732	95,116,807	BAS22_1	SouthFrench1112_1	17.62	7.1
1	87,932,732	95,116,807	SouthFrench3326_2	BAS22_1	18.54	7.1

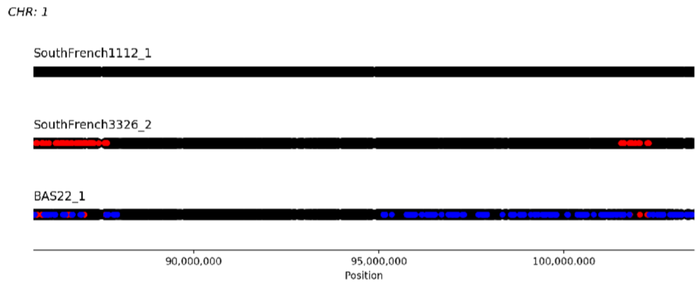

b)

Chr	Start	End	Ind1	Ind2	Score	Length
1	58,466,016	63,501,940	HGDP00807_1	HGDP00810_1	27.51	7.67
1	63,498,807	79,065,052	HGDP00807_1	HGDP00810_2	40.76	14.29

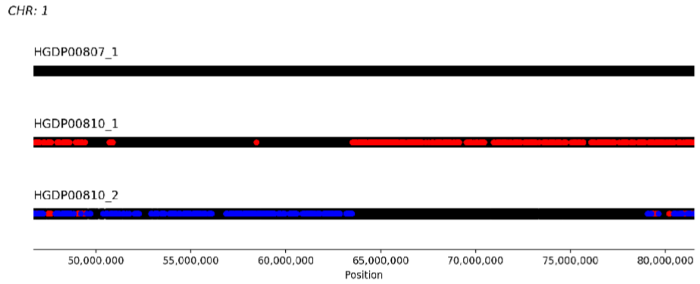

c)

Chr	Start	End	Ind1	Ind2	Score	Length
12	52,931,547	58,839,960	BAS28_1	BAS27_1	13.83	6.37
12	53,315,157	66,115,059	HG01699_1	BAS28_1	22.63	11.5

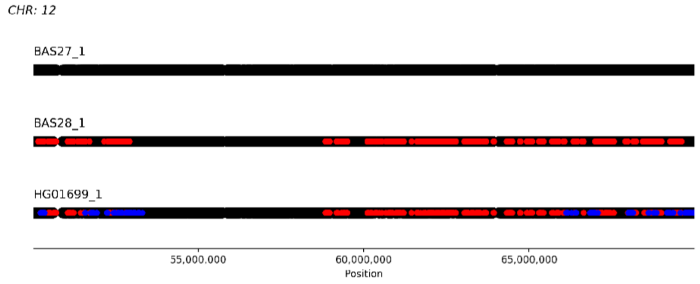

d)

Chr	Start	End	Ind1	Ind2	Score	Length
12	65,921,662	76,538,614	HGDP00805_1	HGDP00806_2	31.56	13.8
12	66,522,359	70,448,946	HGDP00805_1	HGDP00810_2	15.59	6.0
12	66,522,359	70,448,946	HGDP00806_2	HGDP00810_2	13.9	6.0
12	70,458,817	76,538,614	HGDP00805_1	HGDP00810_2	10.8	6.57
12	70,458,817	78,490,195	HGDP00806_2	HGDP00810_2	17.41	8.7

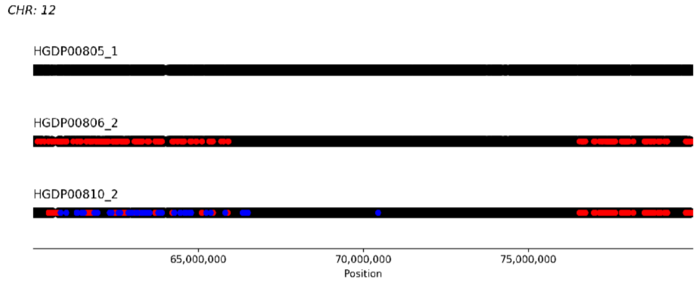

e)

Chr	Start	End	Ind1	Ind2	Score	Length
12	76,475,750	81,145,811	HGDP00524_2	HGDP01377_1	6.82	6.24
12	76,538,614	83,274,174	HGDP01375_2	HGDP01377_1	10.01	6.69
12	77,107,258	83,274,174	HGDP01375_2	SouthFrench3326_1	7.41	6.3
12	77,107,258	83,497,999	HGDP01377_1	SouthFrench3326_1	7.14	6.64
12	78,490,195	89,445,539	HGDP01368_1	HGDP01375_2	14.95	7.88
12	80,158,701	91,723,761	HGDP01368_1	SouthFrench4018_1	18.57	7.76

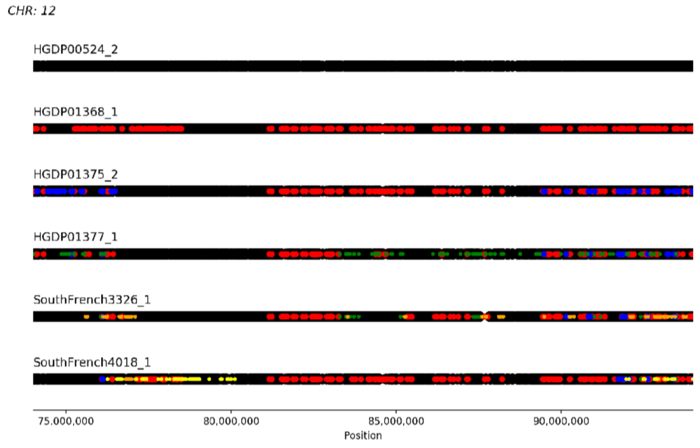

Supplementary Figure S11. Clusters of IBD blocks representing some of the typologies found. For each cluster we show on top the list of IBD segments involved. The label of the individuals of each IBD block is followed by a number (1 or 2) indicating the chromosome (from the two homologous chromosomes) responsible for the IBD. Bellow it there is a representation of the values of the SNPs in all the chromosomes involved on that cluster. For the first chromosome all SNPs are painted in black, for the second they are in black if they present the same allele than the first homologous chromosome and in red otherwise. For each subsequent chromosome new colors are added if a SNP presents a value not encountered before. This creates a visual pattern that allows us to easily

identify segments that are identical. a) A perfect triangulation with 3 IBD segments comprising 3 individuals. The differing length of the IBDs segments can be seen as the result of a shorter identical region in the third individual. b) Two IBD segments between one individual and two consecutive regions on both chromosomes of another individual. A haplotype estimation error is probably behind this pattern and so no third IBD segment should be expected to close the triangulation. C) An open triangulation. The chromosome view allow us to see that the missing IBD segment is shorter than the one 6.37 cM long. Being shorter than 6 cM, it was excluded from the filtered list. D) A multiplication of IBD segments in an apparent simple triangulation brought about by a single mutation/genotyping error shown as a blue point in the third individual. E) A complex cluster that generates various IBD segments with length slightly over 6cM.

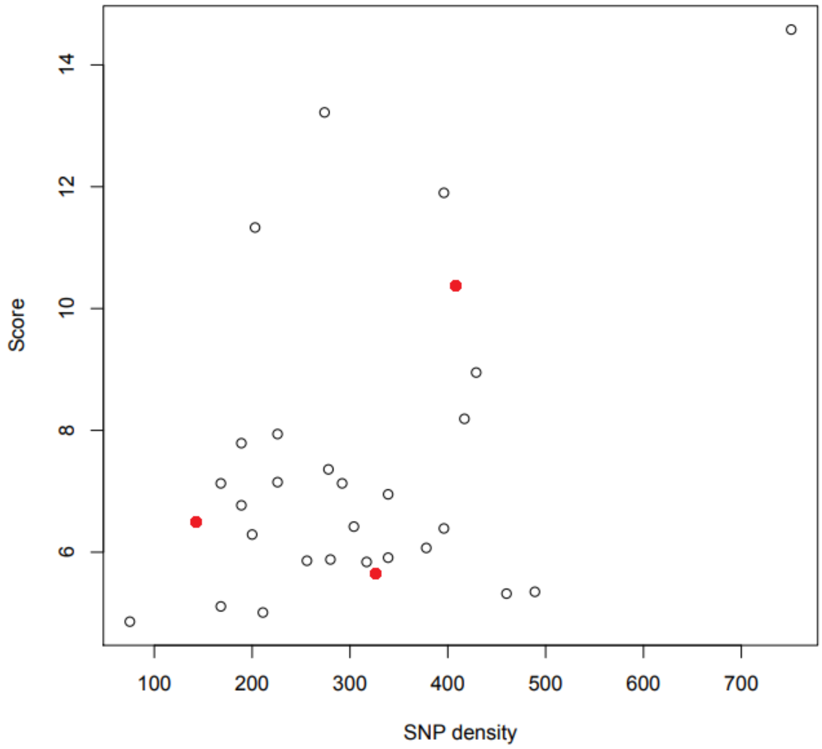

Supplementary Figure S12. SNP density in the Medieval (>2cM) IBD segments plotted against the refineIBD score; three IBD fragments found to be shared with three present-day Lithuanians and susceptible of being false-positives are marked in red. There is a weak positive correlation between higher scores and higher number of SNPs supporting each IBD fragment (R^2=0.165, p=0.0135).

Supplementary Table S1. European populations used in the IBD analysis.

Population	Individuals
French	61
Spanish	53
Basque	29
Sardinian	27
Russian	22
Italian_North	20
Hungarian	20
Greek	20
Orcadian	13
Icelandic	12
Sicilian	11
Norwegian	11
Romanian	10
Lithuanian	10
Estonian	10
English	10
Czech	10
Croatian	10
Bulgarian	10
Belarusian	10
Ukrainian	9
Maltese	8
Cypriot	8
Finnish	7
Albanian	6
Spanish_North	5
Italian_South	5
Scottish	4
Canary_Islander	2

Supplementary Table S2. IBD blocks longer than 6cM between pairs of European individuals.

Supplementary Table S3. IBD blocks longer than 2cM between the Medieval and modern individuals.
